# Brown bear skin-borne secretions display evidence of individuality and age-sex variation

**DOI:** 10.1038/s41598-023-29479-y

**Published:** 2023-02-23

**Authors:** Melanie Clapham, Abbey E. Wilson, Candace L. Williams, Agnieszka Sergiel

**Affiliations:** 1 Department of Geography, University of Victoria, 3800 Finnerty Road, Victoria, BC V8P 5C2 Canada; 2 Department of Veterinary Biomedical Science, University of Saskatchewan, 52 Campus Drive, Saskatoon, SK S7N 5B4 Canada; 3 Conservation Science and Wildlife Health, San Diego Zoo Wildlife Alliance, 15600 San Pasqual Valley Road, Escondido, CA 92027 USA; 4 Institute of Nature Conservation, Polish Academy of Sciences, Adama Mickiewicza 33, 31120 Krakow, Poland

**Keywords:** Behavioural ecology, Chemical ecology

## Abstract

Scent originates from excretions and secretions, and its chemical complexity in mammals translates into a diverse mode of signalling. Identifying how information is encoded can help to establish the mechanisms of olfactory communication and the use of odours as chemical signals. Building upon existing behavioural and histological literature, we examined the chemical profile of secretions used for scent marking by a solitary, non-territorial carnivore, the brown bear (*Ursus arctos*). We investigated the incidence, abundance, and uniqueness of volatile organic compounds (VOCs) from cutaneous glandular secretions of 12 wild brown bears collected during late and post-breeding season, and assessed whether age-sex class, body site, and individual identity explained profile variation. VOC profiles varied in the average number of compounds, compound incidence, and compound abundance by age-sex class and individual identity (when individuals were grouped by sex), but not by body site. Mature males differed from other age-sex classes, secreting fewer compounds on average with the least variance between individuals. Compound uniqueness varied by body site and age for both males and females and across individuals. Our results indicate that brown bear skin-borne secretions may facilitate age-sex class and individual recognition, which can contribute towards further understanding of mating systems and social behaviour.

## Introduction

Chemical signalling facilitates both short and long-range communication in the majority of taxa. While social species frequently integrate concurrent modes of communication (visual, auditory, and chemical)^1,2^, more solitary species tend to prioritise chemical signals as “bulletin boards'' that broadcast long-lasting messages in the absence of the signaller^3,4^. Chemical signals are intended to influence the behaviour of conspecifics^5^, usually to obtain or defend a resource and in sexual advertisement^6,7^. Signalling sexual receptivity and dominance should be equally important for solitary and social species to gain fitness benefits^8^. It has been suggested that the function of chemical signalling may be density dependent^9,10^, and vary by the age, sex or social rank of the signaller^11–14^. The seemingly complex role of chemical communication in the social systems of solitary species is an ecological knowledge gap that requires further exploration.

Odour originates from excretions and secretions and its chemical complexity in mammals translates into a diverse mode of signalling^15–19^. Some species have evolved modified glands in particular areas of the skin, such as the face, flanks and appendages, which are selectively used to scent mark objects, self-anoint or allo-mark^4,18,20,21^. Glandular secretions from different body sites may convey different information by emitting different compounds (incidence) and/or varying proportions of the same compounds (abundance)^4,22–24^, also known as digital and analog coding of information, respectively^25^. Coding for sex and age within odour has been found in both compound incidence and abundance^26–28^ and individual chemical profiles can be discriminated by differences in relative abundance of compounds^29–31^. In solitary species, the ability to recognize individual odours can aid mate identification^32,33^ and discrimination between familiar and unfamiliar conspecifics^29^, with differences in associated risk. Identifying how information is coded can not only facilitate understanding of the mechanisms behind chemical signalling in solitary mammals but also elucidate social function and broader ecological consequences.

Ursids are wide-ranging and largely non-territorial mammals who are believed to rely heavily on chemical signals to communicate with conspecifics. They are solitary but form aggregations at productive feeding sites, around oestrous females, and to play^34–37^. Evidence suggests that bears have evolved a highly developed olfactory system to process odours, enlarged or modified glands to deposit odour, and behavioural strategies to supplement the olfactory signal and reduce energetic costs^38–44^. Ursids deposit scent marks in the form of glandular secretions and urine onto the ground and onto objects in their environment, mainly trees^40,41,45–48^. Specific postures have evolved in some ursids that are thought to deposit cutaneous (skin-borne) scent from different areas of the body, the most dominant being bipedal back (dorsal) rubbing, but also, flank and head rubbing^49–51^, and pedal marking^41^. Little investigation has been conducted into the use of urine and faeces as chemical signals for most ursids, despite observations of urination during pedal marking and rubbing^51,52^. Urine is used by some mammals for chemical communication, including giant pandas (*Ailuropoda melanoleuca*)^53,54^ and wolves (*Canis simensis; C. lupus*)^55,56^. Giant pandas and brown bears (*Ursus arctos*) also possess anal glands, which giant pandas use extensively for chemical communication^30,57^. Less is known about the use of anal gland secretion (AGS) in other ursids, although Rosell et al*.*^28^ found that brown bear AGS may code for sex. Compound volatility can also help to interpret function; brown bear^28^ and giant panda^30^ AGS compounds were predominantly those of low volatility (molecular weight > 300), advantageous for delayed communication in species with large home ranges such as bears. Chemical profiles from AGS and excretions may code for varying biological attributes compared to skin-borne secretions.

Recent histological investigations into sebaceous and apocrine glands in the dermis of brown bears dorsal region found seasonal variation in their size and volume in males^43,44^. Other studies found male-specific compounds to be present in pedal secretion^41^, but not AGS^28^. These studies form the only known chemical analyses of brown bear glandular secretions described in the literature. Behavioural evidence suggests that the function of chemical signalling in this species is to communicate dominance between males and/or attracting mates^10,38,58^. However, with still insufficient chemical evidence, signal content of cutaneous scent remains uncertain. Examining whether scent chemical composition varies would be the first step in determining if these signals code for different information according to the body region, or age and sex of the signaller, similar to age-related changes in temporal gland secretion of male Asian elephants (*Elephas maximus*)^12^. Chemical investigation in combination with existing behavioural evidence is therefore vital to address proximate and ultimate questions regarding chemical signalling in brown bears.

In this study, we analysed volatile organic compounds (VOCs) from cutaneous glandular secretions of wild brown bears collected during late and post-breeding season to detect variation in chemical composition based on a) age-sex class, b) body site, and c) individual identity. We explored signal content by assessing VOC profile composition in the number of compounds, their incidence and abundance, as well as compound ‘uniqueness’ across a range of conditions (e.g., male and female within ‘sex’). We predicted that (1) chemical profiles would show age-dependent sexual dimorphism^28,38,59,60^; (2) mature male chemical profiles would show more variation compared to mature females^61,62^; (3) secretion from the dorsal region of the skin would show more variation than other body sites^43,44,49^; and (4) chemical profiles would encode information on individual identity^30,61,63^. By investigating the mechanisms of chemical signalling in a solitary, non-territorial carnivore, we can address knowledge gaps in aspects of ursid ecology such as social dynamics and mating behaviour. In addition, results can inform the development of biomarkers for wildlife monitoring based on the association between chemical signatures and biological attributes (see^64,65^).

## Methods

### Site description

Sampling was conducted in south-eastern Alaska, USA, across an area of 3191 km^2^ along the Yakutat forelands (59°17′24′′ N, 138°53′14′′ W). The study site stretches from the Pacific Ocean to the west and extends 100 km east to border glaciers and mountains to the north and east. The majority of the site is within the United States Forest Service Tongass National Forest. Habitats range from intertidal flats, wetland shrubs and herbaceous vegetation, to Sitka spruce (*Picea sitchensis*) and western hemlock forests (*Tsuga heterophylla*). Food resources available to brown bears within the study area during late summer, include coastal strawberry (*Fragaria chiloensis*), small pelagic fishes [e.g., surf smelt (*Hypomesus pretiosus*)] and all five species of Pacific salmon (*Oncorhynchus* spp.). Density estimates for brown bears at the site are 98.8 ± 8.2 bears/1000 km^2^^66^.

### Capture protocols

Brown bears were captured, immobilized, and fitted with GPS collars for a different study from July 2009 until September 2014 (see ^66^). Capture and handling protocols were approved by Alaska Department of Fish & Game’s Division of Wildlife Conservation Institutional Animal Care and Use Committee (protocol 2013-028) and were in line with procedures outlined by the American Society of Mammalogists^67^. Age (years) of the bears captured was identified by extracting a premolar tooth and conducting cementum analysis^68^. For further details on capture methodology (including drugs administered and equipment used) see Crupi et al*.*^66^.

### Scent collection protocols

Samples for this study were collected from bears from 24 July to 23 September 2014. Twelve bears were captured in total during this sampling period, which included two mature males (15 and 19 years old), two mature females (14 and 19 years old), six young (independent subadults) males (3–4 years old) and two young females (4 and 6 years old; Table 1).

**Table 1:** Wild brown bears sampled in south-eastern Alaska, USA.

Individual ID	Sex	Age (years)	Age class^a^	Sample *n*
Y861	F	14	Mature	8
Y866	F	19	Mature	8
Y865	F	6	Young	4
Y867	F	4	Young	8
Y742	M	19	Mature	8
Y868	M	15	Mature	4
Y743	M	4	Young	4
Y744^b^	M	3	Young	2
Y860	M	4	Young	8
Y862	M	4	Young	8
Y863	M	3	Young	8
Y864	M	3	Young	8

^a^Young   < 10 years, Mature   ≥ 10 years.

^b^This individual was excluded from the ‘individual’ treatments due to an incomplete dataset.

Samples were collected from four body sites: cheek (n = 20), flank (n = 20), hump (muscle between the shoulder blades; n = 19), and pedes (between the digits, plantar surface of the paw, any paw and digits; n = 19). These body site sample collection locations were selected based on behavioural evidence of scent marking postures, and represent distinctive body parts most commonly rubbed against, or at the base of, marking trees^49^. Likewise, we sampled all age-sex classes due to the varied behaviour of each at marking sites, namely selection of different scent marking postures^49,69^ and frequency of marking^38^.

To collect a scent sample, the hair was parted and medical-grade sterile cotton gauze was manually rubbed against the surface of the skin. The sample was then placed into 4 mL glass vials with Teflon PTFE-lined caps. This procedure was repeated per bear, sampling an adjacent patch of skin from the same body site and stored in a separate glass vial to collect a replicate sample. For sampling between the toes (pedes), the replicate sample was taken between the adjacent toes to the initial sample. Powderless nitrile gloves were worn at all times during scent collection. Gloves were changed or cleaned with Distel™ disinfectant wipes before sampling different body locations on each bear. Glass vials were frozen at −20 °C as soon as possible on the day of collection. Samples were shipped from Alaska, USA to British Columbia, Canada for analysis in 2015. Samples were kept frozen during transportation using 4 Nu-Ice Marine Series Cooler packs (−16 °C freezer charge) placed into a Yeti^®^ Hopper™ cooler bag. Samples were then transferred to a laboratory freezer (−75 °C) prior to analysis.

### Chemical analyses

All samples (n = 78) were extracted and analysed in 2017 by the British Columbia Ministry of Environment North Road Analytical Laboratory (Victoria, BC, Canada). Volatile compounds were extracted from sample gauze using 10 mL of methanol in a glass headspace vial (Perkin Elmer). Samples were hand-shaken for 5 s to ensure dispersion in the solvent. The vials containing the methanol and the sample gauze were introduced via an automated headspace sampler (Turbomatrix 110 Trap with helium carrier gas) into a gas chromatograph mass spectrometer [GCMS; Perkin Elmer Clarus 500, using a Zebron ZB-Waxplus Column (30 m, 0.25 mm ID, 0.25 µm film thickness)]. The headspace vial containing the extract was heated to 80 °C and agitated for 15 min so that the analytes of interest migrated into and formed an equilibrium with the headspace in the vial. The headspace was sampled with the needle at 90 °C and was allowed to desorb for 0.5 min. The headspace trap and purge as well as the GCMS settings were optimized using domestic dog (*C. familiaris*) hair to maximize sensitivity and accuracy of the instrument. Briefly, the GC oven was kept at 60 °C for 1 min, increased to 85 °C at 3 °C/min, from 85 to 170 °C at 8 °C/min, from 170 to 250 °C at 20 °C/min and then held at 250 °C for 8 min, resulting in a total run time of 31.96 min. The MS was operated in scan mode, using electron impact ionization and a scan range from 50 to 500 *m/z*.

### Data processing

Initially, data files for each sample were converted to a common data format (.cdf files) using OpenChrom Software (Community Edition 1.3.0), which allows for the analysis and visualization of native data files from different mass spectrometry systems. One file failed to convert (pede sample from Y862) and was removed, resulting in n = 77 samples analysed. Next, files were converted to Agilent data files (.d files) using Agilent GCMS Translator software (Agilent Technologies, Santa Clara, CA) in order to utilize Agilent software workflows. We conducted deconvolution on chromatogram data in order to improve detectability, identification, and visualization of volatile organic compounds in samples using Agilent MassHunter Qualitative Analysis (for GCMS) Workstation Software (signal to noise ratio threshold 0; absolute height 500 counts; absolute area 5000 counts) in conjunction with the Wiley Registry 10th Edition/NIST 2012 Mass Spectral Library^41,57,63^. Data files for each sample were then converted to compound exchange format (.cef files) and transferred to Agilent Mass Profiler Professional Software in order to align detected peaks across all samples^65,70^. Peak alignment allowed us to determine the presence and absence of compounds as well as the total number of compounds within and across samples. Only 24 out of 254 compounds detected could be identified with a reliable match factor (> 80) to the library database, which are instead listed as mass@retention time (sensu^41^). Therefore, identified compounds were not removed from the detected peak list. Compounds were further filtered by relative abundance, occurrence and contribution to the volatile profile (described below), which resulted in 4 identified compounds. Similar to previous studies, tentatively identified compounds were not confirmed with known standards^28,30,71,72^.

### Data analysis

We examined whether biological attributes explained variation in VOC profiles of brown bears using R statistical software (version 3.6.3)^73^. Replicate samples were included in analyses to ensure all potential volatile compounds were collected (as in^74,75^). This resulted in strict criteria for each compound to be included, as the relative abundance and presence of each compound had to meet certain thresholds in both samples, rather than using these metrics for one sample or an average across both samples. Compounds with an abundance of zero were replaced with half the value of the minimum abundance compound to account for the uncertainty of true zeros or non-detections (below the detection limit)^76,77^. The abundance of each compound was divided by the total abundance of all compounds in a sample to calculate the relative abundance of each compound to the overall scent profile^72^. We transformed the dataset using the square-root transformation to help achieve normality.

To determine if data were homogeneous, we first measured the variance across the different groups: (1) age class, (2) sex, (3) age-sex, (4) body site (nested by individual within sex), and (5) individual (nested in sex) (Supplementary Table S1). To evaluate how VOC profiles varied we used permutational multivariate analysis of variance (PERMANOVA) with Bray–Curtis (compound occurrence and relative abundance) and Jaccard (compound presence/absence) distance matrices, respectively, with 10,000 permutations (*vegan::*adonis^78^), and only included compounds that occurred in at least 5% of all samples (4/77 samples)^27,72^. We initially tested by the aforementioned groups, and subsequently for each age-sex class by (6) body site (nested within individual), and (7) individual, which reduced comparative sample size further but was used for additional inference.

Differences in variance for both compound incidence and abundance was observed, and given the unbalanced nature of the dataset, we randomly subsampled the young male age-sex class to include two individuals (as observed by the other age-sex classes) and found no significant changes to PERMANOVA outcomes (Supplementary Table S2). We therefore report results from the balanced dataset (n = 8 individuals (Y866, Y861, Y867, Y865, Y864, Y743, Y742, Y868), n = 52 samples) only for PERMANOVA analyses.

Beta-diversity was visualized using non-metric multidimensional scaling (nMDS) by generating distance matrices from the GCMS data (*vegan::*vegdist). Based on the goodness of fit R^2^ (0.987) and stress (0.13), three dimensions were used for the nMDS. Finally, compounds of interest were identified using similarity percentages (SIMPER; *vegan::*simper) when compounds contributed to the variation ≥ 2.0%^79–81^. Each compound identified by SIMPER was analysed using the previous model and evaluated using analysis of variance (ANOVA) to determine compounds that varied with respect to mean relative abundance (*stat* package^82^). All data are expressed as the mean ± standard deviation (SD) and considered significant if P < 0.05, unless otherwise stated.

To explore compound uniqueness, we determined the presence and absence of compounds using Agilent Mass Profiler Professional Software and classified compounds according to their presence within and across conditions. Each of the nine treatments contained 2–11 conditions (e.g., treatment ‘sex’ = two conditions: ‘female’, ‘male’), with sample sizes varying per condition (Table 2). Each condition contained samples from ≥ 2 individuals. We then created an ‘individual’ treatment where each individual bear was classed as a separate condition. VOCs were grouped into three categories (1) ‘unique’: present in 100% of samples within *only one* condition and absent in all other conditions, (2) ‘signature dominant’: present in 100% of samples in *at least one condition* but may be present in fewer samples in other conditions, and (3) ‘dominant’: present in 100% of samples within *more than one* condition, and may be present in fewer samples within other conditions. To be included in analyses, compounds had to occur in 100% of samples for at least one of the two (or more) conditions per treatment. Therefore, compounds could occur in more than one condition. For all treatments (except ‘individual’) this controlled for outlier compounds which could have been a result of individual variation in chemical profile or other biotic factors, but were not representative of a specific condition as a whole. Individual Y744 was not included in the ‘individual’ analysis due to an incomplete data set.

**Table 2:** Number of samples and conditions in each treatment comparison for analyses of compound uniqueness.

Treatment	Sample *n*	Conditions	*n* per condition
Sex	77	2	28 (female) 49 (male)
Age class^a^	77	2	49 (young) 28 (mature)
Female; age class	28	2	12 (young) 16 (mature)
Male; age class	49	2	37 (young) 12 (mature)
Female; body site	28	4	7 (cheek) 7 (flank) 7 (hump) 7 (pedes)
Male; body site	49	4	13 (cheek) 13 (flank) 12 (hump) 11 (pedes)
Female; age class; body site	28	8	3 (young; cheek) 4 (mature; cheek) 3 (young; flank) 4 (mature; flank) 3 (young; hump) 4 (mature; hump) 3 (young; pedes) 4 (mature; pedes)
Male; age class; body site	49	8	10 (young; cheek) 3 (mature; cheek) 10 (young; flank) 3 (mature; flank) 9 (young; hump) 3 (mature; hump) 8 (young; pedes) 3 (mature; pedes)
Individual	75	11	8 (Y861) 8 (Y866) 4 (Y865) 8 (Y867) 8 (Y742) 4 (Y868) 4 (Y743) 8 (Y860) 7 (Y862) 8 (Y863) 8 (Y864)

^a^ Young   < 10 years, Mature   ≥ 10 years.

## Results

Using 77 wild brown bear samples (n = 12 individuals), we detected 254 compounds (5% incidence) across all samples. Representative total ion chromatograms are shown in Supplementary Fig. S1.

### Number of compounds

**Figure 1:**
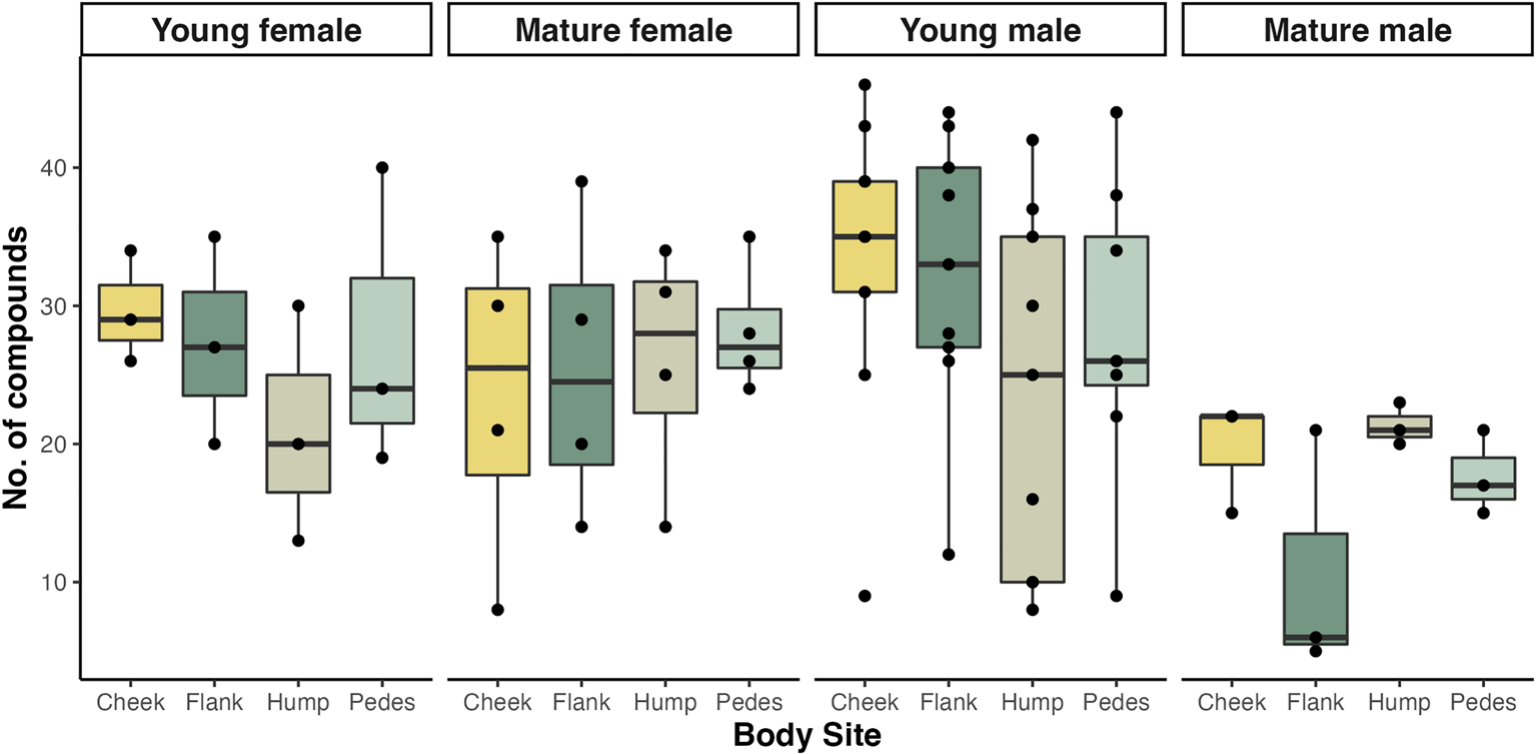
The mean number of compounds (> 0.50% relative abundance) in cutaneous scent samples, split by age-sex class and body site.

The average number of compounds varied by age-sex class (*F* = 7.97, *P* = 0.008, ANOVA; Fig. 1), with samples from adult males having significantly fewer compounds than young males (mature: 17.3 ± 5.9, young: 29.4 ± 11.4, *P* < 0.001, Tukey HSD), mature females (25.8 ± 8.4, *P* = 0.044), and young females (26.4 ± 7.4, *P* = 0.044; Fig. 1). While age was a significant predictor of the number of compounds (*F* = 11.47, *P* = 0.002), and sex was not (*F* = 0.96, *P* = 0.335), we cannot interpret these variables independently due to the significant interaction between age and sex. The average number of compounds did not vary by body site nested in ID and sex (*F* = 1.03; *P* = 0.464), but did vary by individual nested in sex (*F* = 5.69, *P* < 0.001), with significant pairwise differences driven by one individual with fewer compounds (Y743: 10 ± 1.2; Fig. 1).

### Profile variation by compound incidence and abundance

#### Age-sex and individual differences

**Figure 2:**
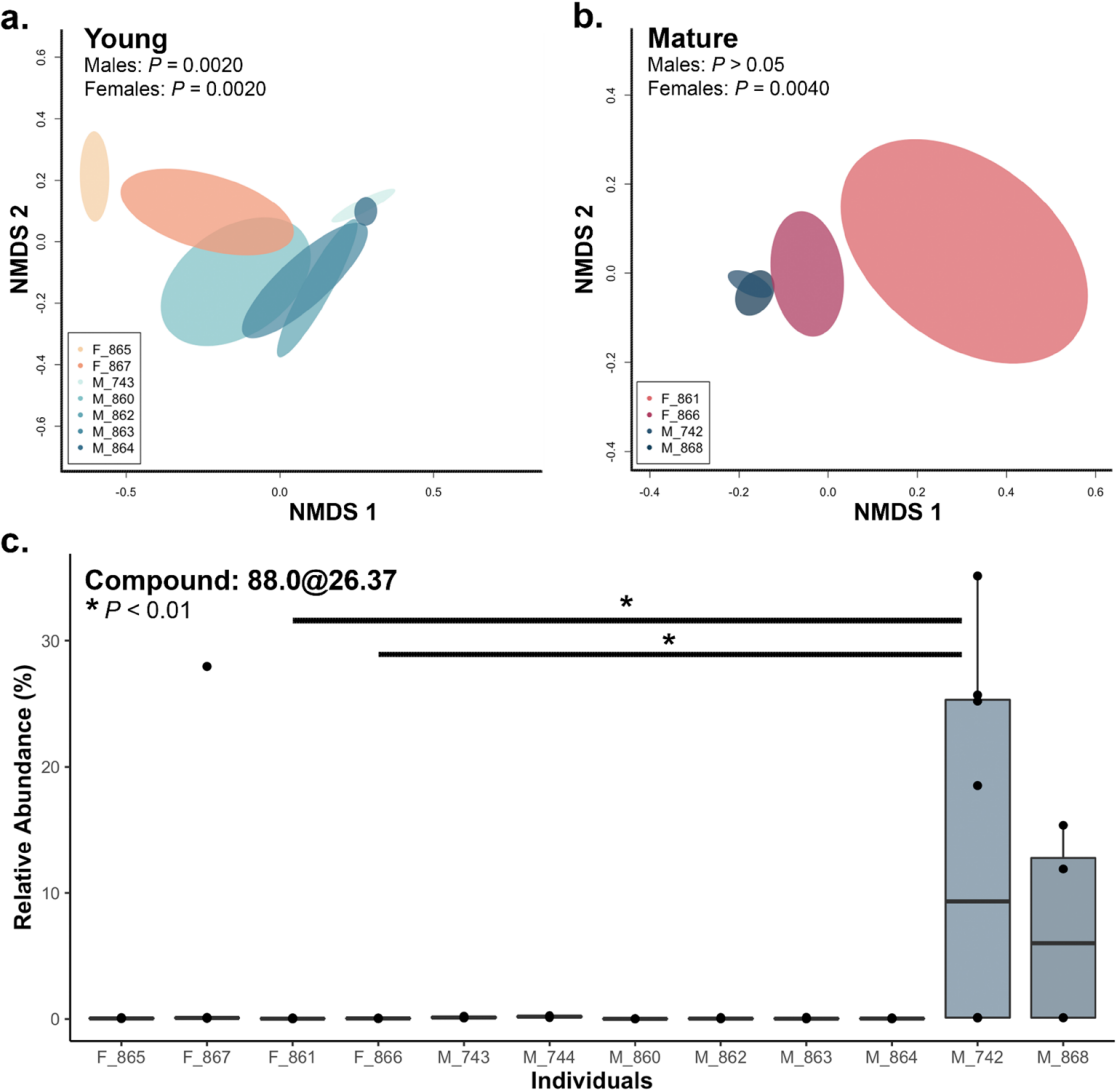
Nonmetric multidimensional scaling (nMDS, Bray–Curtis) analysis displays differences in volatile organic compounds in sample swabs collected from brown bears by age-sex class for (**a**) young bears (stress: 0.10, goodness-of-fit: 0.99) and (**b**) mature bears (stress: 0.090, goodness-of-fit: 0.992) displayed by ellipses depicting 99% confidence interval of ordination object. (**c**) Relative abundance of compound 88@26.37 is found to drive variance between individuals. Asterisk: significant pairwise comparison with Bonferroni correction, both P < 0.01.

Both the incidence and abundance of compounds varied significantly with respect to (1) age-sex class (Table 3) and (2) individuals nested within sex (Table 3). These differences were visualized both overall (Supplementary Fig. S2) and separated by age class to assess age-dependant sexual dimorphism (Fig. 2a,b). We found 23 compounds that contributed to the variation in the volatile profile (≥ 2%) of individuals nested within sex (Supplementary Table S3), and one compound (72.0@1.97) that contributed to the variation of volatile profiles within age class. Of these, we found the relative abundance of only three compounds to be significant across sex with respect to mean relative abundance (88.0@24.87, SIMPER 2.28%, *P* < 0.001; 2,5-dichlorobenzyl alcohol SIMPER 2.09%, *P* < 0.001; 74.0@24.95 SIMPER 2.93%, *P* = 0.022).

**Table 3:** PERMANOVA results using Bray–Curtis and Jaccard distance matrices for analyses of brown bear cutaneous chemical profiles.

Term	Distance	df	Pseudo-*F*	*P* (perm)	Adj. *P* (FDR)
Age	B–C	1	2.253	0.010	**0.017**
	Jaccard	1	1.874	0.014	**0.018**
Sex	B–C	1	2.114	0.015	**0.019**
	Jaccard	1	1.986	0.010	**0.017**
Age*sex	B–C	1	5.076	< 0.001	** < 0.001**
	Jaccard	1	4.089	< 0.001	** < 0.001**
ID (sex)	B–C	4	2.766	< 0.001	** < 0.001**
	Jaccard	4	2.297	< 0.001	** < 0.001**
Body site (ID(sex))	B–C	24	0.986	0.546	0.546
	Jaccard	24	0.977	0.594	0.594
Residuals	B–C	20	0.312		
	Jaccard	20	0.330		

*B–C* Bray–Curtis, *df* degrees of freedom, *P* (*perm*) significant effects < 0.05 with 10,000 permutations, *Adj. P *(*FRD*) adjusted P-value for multiple comparisons (using false discover rate).

Significant values are in bold.

Within age-sex classes, we found significant differences in compound incidence and abundance between individual bears with the exception of mature males (Supplementary Table S4). The differences in variance observed can be illustrated by nMDS, with young males and mature females having larger sample variance and little overlap compared to mature males with smaller variance and a strong overlap of ellipses (Fig. 2). We found 12 compounds that contributed to the variation in the volatile profile within mature bears and 15 compounds that contributed to the variation within young bears (separated by individuals nested within sex) (Supplementary Table S5). For example, 88.0@26.37 was found in higher relative abundance in mature male bears compared to their female counterparts, or young bears, with mature male Y742 displaying the highest relative abundance (Fig. 2c).

#### Body site differences

**Figure 3:**
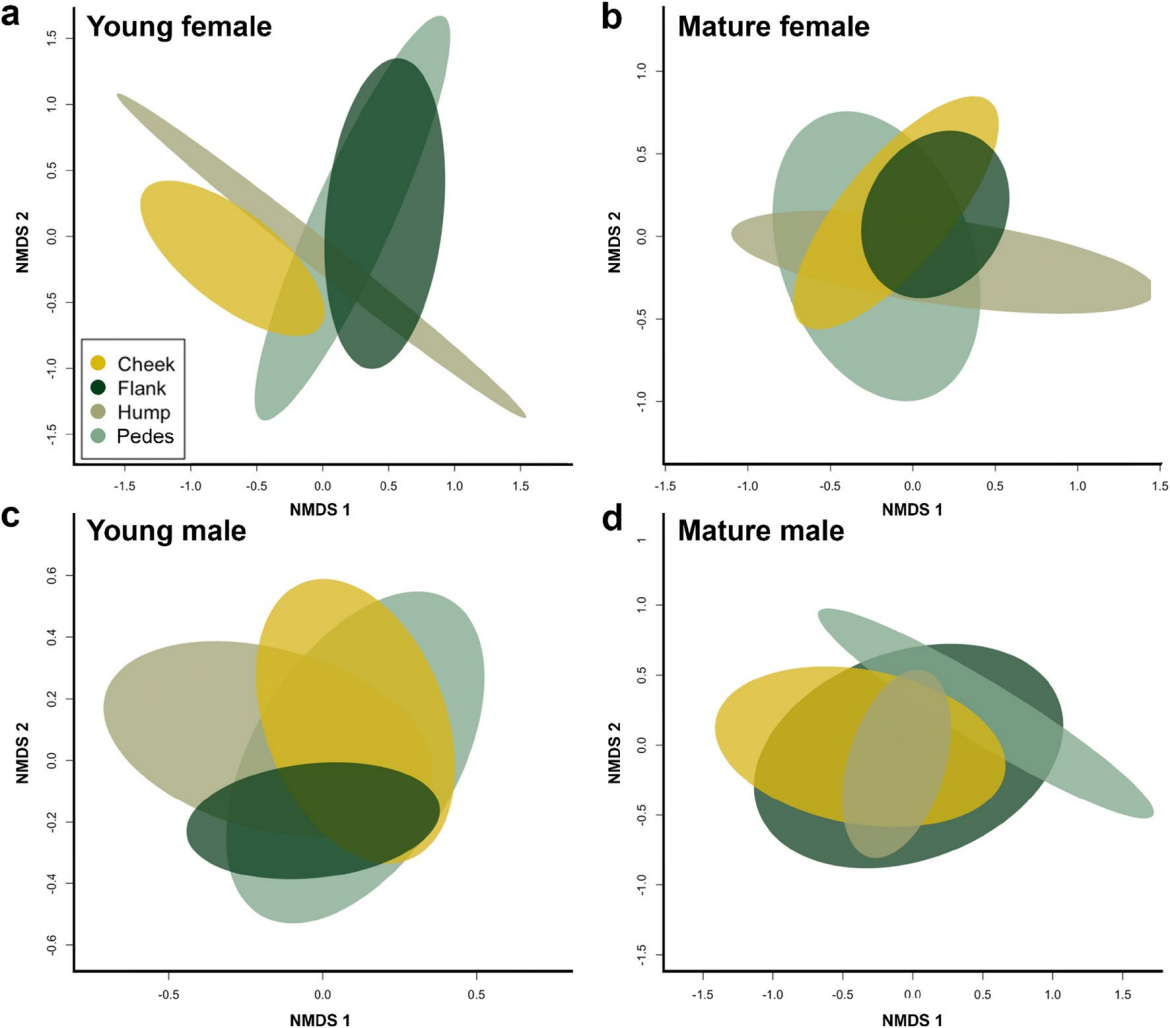
Nonmetric multidimensional scaling (nMDS, Bray–Curtis) displaying differences in body site within age-sex classes, (**a**) young female (stress: 0.14, goodness-of-fit: 0.98), (**b**) mature female (stress: 0.18, goodness-of-fit: 0.966), (**c**) young male (stress: 0.23, goodness-of-fit: 0.945), and (**d**) mature male (stress: 0.14, goodness-of-fit: 0.979) displayed by ellipses depicting 99% confidence interval of ordination object.

We did not find the incidence or abundance of compounds in the volatile profiles to vary across body sites when nested by individual within sex (Table 3), or nested within individual and grouped by age-sex class (Supplementary Table S4). Non-significant body site differences by age-sex class are illustrated by nMDS, showing overlapping ellipses (Fig. 3).

### Compound uniqueness by condition

We found unique (100% of samples within *only one* condition and absent in all other conditions), signature dominant (100% of samples in *at least* one condition, lower % in others), and dominant (100% of samples in *more than one* condition) compounds varied by (1) body site and age in females and (2) body site and age in males. We did not find unique, signature dominant, or dominant compounds to any condition in comparisons between sex only, age only, females by age, and males by age.

#### Body site and age variation in females

Within mature and young female bears (n = 4 individuals): one unique, 21 signature dominant, and 5 dominant compounds were detected in samples across body sites (Supplementary Table S6). Flank secretions collected from young females had the greatest number of signature dominant compounds (11), while cheek secretions collected from young females contained the lowest number of signature dominant compounds (1). One signature dominant compound (72.0@26.59) detected in samples collected from the flank of mature females only occurred in body sites from mature females, while two signature compounds (62.0@2.63 and 92.0@4.53) detected in samples collected from the flank of young females were only found in body sites from young females. Two compounds, 88.0@23.15 and 88.0@22.72 were found across all samples collected from the flank and interdigital secretions of mature females and the cheek secretions of young females. The only condition not to have a signature dominant compound was the hump of mature, female bears. One unique compound (62.0@2.63) was found in secretions collected from the flank of young female bears; this compound did not occur in any other conditions.

#### Body site and age variation in males

Within mature and young male bears (n = 8 individuals): zero unique, 17 signature dominant, and four dominant compounds were detected in samples collected across body sites (Supplementary Table S7). No signature dominant or dominant compounds were found in the secretions of young male bears from any body site, which could be an effect of a higher sample size for this age-sex class, compared to other age-sex classes. Hump samples collected from mature males had the greatest number of signature dominant compounds (9), while cheek and interdigital gland secretions had the lowest (2). Four dominant compounds occurred in mature male samples compared to young males. For example, 72.0@1.96 and 74.0@24.94 were detected across all samples collected from the cheek and flank of mature males but were only present to a lesser extent in young males.

#### Individuals

Zero unique, ten signature dominant, and one dominant compound were found across individuals (Supplementary Table S8). Ten signature dominant compounds were found in five of 11 bears: two mature males (Y742; Y868), two young males (Y743; Y860) and one young female (Y865); however, this varied for each individual. For example, four signature dominant compounds (86.0@25.01, 59.0@1.79, 74.0@25.24, and 74.0@25.37) were detected in samples collected from mature male Y868, while only one signature dominant compound (74.0@24.94) was detected in samples collected from mature male Y742. One dominant compound (72.0@1.96) was found in all samples collected from young female Y865 and mature male Y868. No dominant or signature dominant compounds were found for two mature females (Y861; Y866), one young female (Y867) and three young males (Y862; Y863; Y864).

## Discussion

Odours play a key role in mammalian communication, conveying information about identity, sex, social status, reproductive state, group membership and/or territorial boundaries^15,18,83^. We sought to understand the mechanisms of olfactory communication in brown bears by investigating the chemical components of skin-borne secretions used in scent marking. Although our sample size is relatively small (n = 12 individuals), to our knowledge we are the first to examine the chemical composition of brown bear cutaneous secretions beyond pedal scents and anticipate this as a starting point for further inquiry. Future studies should aim to collect representative samples across age-sex classes equally, although this can be difficult with wild bears due to the nature of field capture. Likewise, our study would have benefitted from the collection of control samples to easily filter potential contaminants and future studies should follow recent advancements in sample collection and analysis methods^84,85^.

### Chemical profiles code for age-sex class

We found strong evidence for our prediction of age-dependent sexual dimorphism in VOC profiles of coastal brown bears. Age-sex class was a significant predictor of the average number of compounds and compound incidence and abundance. The two other studies that assess compound variation in secretions of wild brown bears found evidence that pedal scent and AGS code for sex, through sex-specific compounds^41^ and the relative abundance of compounds^28^. Here, we present evidence that indicates the importance of an interaction between age and sex in skin-borne secretions. Age-related differences in chemical profiles have also been found in giant pandas^30^, red deer (*Cervus elaphus*)^86,87^, Eurasian otters (*Lutra lutra*)^27,88^, mandrills (*Mandrillus sphinx*)^75^, and white rhinos (*Ceratotherium simum*)^89^. In addition, behavioural indications of age signals in excretions and secretions have also been found in black rhinos (*Diceros bicornis*)^90^ and giant pandas^91^.

We found that changes in the average number of compounds between age-sex classes was driven by adult males. Age-related differences in chemical profiles have been shown to be hormonally regulated in mammals^92^, which with physiological maturity, affect the chemical constituents of secreted scent^27,75^. In this study, a single compound contributed significantly to age-related differences in chemical profiles. Investigations into the influence of symbiotic microbes on mammalian odours have found variation in bacterial communities by age class. Hormones can drive shifts in microbial composition and microbes can transform hormones (reviewed by^93,94^). This dynamic relationship can lead to differences in microbial composition related to age and reproductive cycle, thus driving changes in chemical composition associated with each condition (reviewed by^95^). This phenomenon has been observed in striped hyenas (*Hyaena hyaena*), where scent pouch composition and structure varied by age-class, with adults possessing a core suite of microbiota and younger animals showing more variation^96^. Age determination from chemical signals could have fitness advantages for both young and mature bears. With some variation, brown bears reach sexual maturity at ~ 5 years of age for both males and females^97^. However, age is correlated with body size in males, and females select larger males as mates^98^, therefore older males have greater reproductive success^99^. Likewise, females between nine and twenty years old produce the most offspring^100^. Age determination could function in mate selection and competitor assessment for mature bears, and may reduce risk to younger bears through honest signalling (sensu^101^). For example, in Asian elephants, the musth of young males varies from that of mature males in composition, and is thought to convey a non-threatening chemical message of naivety to avoid conflict with older males^12^. A signal of subordinance (low competitive ability) could be advantageous for brown bears in a similar way. As a solitary carnivore that exhibits breeding and foraging aggregations, and female matrilineal assemblages^102^, subordinate bears may gain benefits from signalling low competitive ability if the risk of accessing important habitat in close proximity to other bears is reduced especially at high densities (akin to behavioural signals of submission during direct encounters, such as ‘face away’ postures^37^).

Brown bears in this study were divided into age classes based on the demographics of the dataset; the difference between the eldest bear in the class ‘young’ and the youngest in the class ‘mature’ was eight years. This ensured a representative division according to life stage, and we believe reduced error in assignment concerning young adult bears (5–6 years of age). Comparative studies should be aware of the potential for varying results based on age classification and between age groups in the analysis of age-related chemical signals (sensu^96^).

Contrary to our prediction that mature male profiles would show more variance than mature females, mature male bears were the only age-sex class where compound incidence and abundance did not vary significantly between individuals, and mature male individual profiles showed less variance than mature females and other age-sex classes. Mature male brown bears are considered to be the dominant age-sex class based on behavioural observations^103,104^, and have been shown to engage more in scent marking than other age-sex classes, including frequency and time investment^48,49^. As skin-borne chemical profiles in male brown bears appear to encode a signal for age (see above), which for other species (Asian elephant) conveys an honest signal of competitive ability^12^, perhaps a mature male chemical signature—a signal of high competitive ability—is also present here. We are unable to develop this hypothesis further due to low sample size of mature males in our study (n = 2 individuals, 12 samples), however behavioural evidence indicates adult male-to-male signalling via scent marking in some populations^38,48^. Likewise, the scent profile of high-ranking female spotted hyena (*Crocuta crocuta*) contains an ‘olfactory badge of status’^105^. Further work is needed to test this hypothesis on a larger sample size of adult male brown bears.

### Chemical profiles code for individuality

Chemical signals of individuality are well documented in terrestrial mammals, including mustelids^72,106^, rodents^107–109^, primates^75,110^ and carnivores^31,63,111^. While we found no unique compounds across individual brown bears, our prediction that profiles code for individual identity was supported: compound incidence and abundance varied by individuals within sex and when individuals were grouped by age-sex class. Individual compound uniqueness also varied according to individual bears. Mammalian chemical profiles are known to be complex mixtures of a variety of chemical components that vary in incidence and abundance, resulting in distinctive odours^112–115^. Adding additional complexity, we found that compound uniqueness showed interindividual variation, with some compounds expressed in all samples from one individual but absent from another. However, we did not find consistent compound uniqueness specific to each individual in the study.

The ability to discriminate individuals based on their chemical profile would provide considerable benefits for species that are able to retain this information for later use, and modify their behaviour in such a way that reduces risk and/or provides a benefit^108^. The presence of individual scent signatures does not necessitate their use in individual recognition, nor that they have evolved for this function. Nonetheless, if individual recognition contributes to fitness, individually-distinctive odours should be used by other animals to gain information on conspecifics and their use as a chemical signal would evolve. Individuals that advertised their distinctive odours (e.g., through scent marking) would then have a competitive or reproductive advantage, especially if their odour also contains coded information on age and sex. Bears are thought to possess the ability to recognise previous mates^116^, kin^102,117^, and other conspecifics^118,119^, and scent marking has been proposed as a method of signalling dominance and mate attraction^10,38^. Indeed, Morehouse et al*.*^120^ recently found a positive relationship between tree rubbing and reproductive success for both male and female brown bears, and Hansen et al*.*^119^ found that familiarity between female bears was important for home range settlement, which they suggest is facilitated in part by scent cues. Although brown bears are not a gregarious species, they do have some ecological traits (e.g., breeding and feeding aggregations, overlapping home ranges) that lead to conspecific interactions and result in a social hierarchy. Chemical signals that encode individuality could facilitate individual recognition within these social contexts.

### Body site variation and compound uniqueness

We found no body site-specific differences in the average number of compounds, and no significant variation in compound incidence and abundance between body sites when nested within individuals and grouped by age-sex class.

Compound uniqueness varied by body site and age within sex, and samples from the dorsal (hump) region of mature males showed the highest level of compound uniqueness across males; giving partial support to our prediction of increased variation of hump samples compared to those taken from other body sites. In behavioural studies, dorsal (back) rubbing is a core marking posture, particularly for adult males^49,121,122^. Histological and histochemical analyses of the hump area of male bears have shown that sebaceous glands are enlarged and produce more oily secretion prior to and during the breeding season for intact males, influenced by testosterone concentration^44^. Similar was found for apocrine glands during the breeding season, indicating that both sebaceous and apocrine glands in the back of male bears vary by season and reproductive status^43^. As our samples were collected towards the end of- (July) and post-breeding season (August/September), chemical profiles may vary to those during the breeding season, especially in relation to body sites of male bears. While our samples were collected opportunistically and therefore, we could not control the time of year of collection, future studies should compare these results to samples taken during the breeding season, for both sexes. Histological and behavioural analyses in combination with the results presented here, provides a body of evidence that supports back rubbing as a focal method of chemical signalling for male brown bears.

It remains unclear why bears rub different body sites against trees (and other objects). Giant pandas are thought to mark urine using the handstand posture to deposit scent higher on trees, which communicates size and therefore competitive ability^3^. Our results indicate that while different body site secretions may contain different compounds, very rarely are they unique to that body site across either individuals or age-sex classes. In addition, the lack of variation across body sites within chemical profiles poses the question: why are brown bears using multiple body sites when marking with cutaneous secretions? One explanation that we propose is that these glands are relatively small, compared to highly specialised scent glands such as anal glands or sacs, and produce a smaller volume of secretion compared to urine or faeces excretion. Indeed, Alberts^123^ proposed that by marking on elevated surfaces, signalling animals increase the active space of the scent mark and that for certain species, hair may facilitate the distribution of scent over a larger area. Therefore, marking with multiple body sites may increase the surface area of the overall scent mark, but not necessarily provide different coded information for brown bears, at least outside of the breeding season.

The surface may also influence the body site used, e.g., pedal marking the ground^41^ compared to, or in combination with, tree marking. We suggest further analyses of brown bear cutaneous scent focus on hump and pedal body sites only, to reduce potential redundancy. An exception to this could be examining the flank secretions of female bears (see “Results”)^49^ in combination with the hump secretions of males during the breeding season.

## Conclusion

We explored signal content and found that skin-borne VOC profiles coded for age-sex class and individual identity in brown bears, when individuals were both nested in sex and grouped by age-sex. A suite of compounds varied in both incidence and abundance within chemical profiles, according to these attributes. Mature males differed from other age-sex classes, secreting fewer compounds on average with the least variance between individuals. Compounds were rarely unique to specific ages, sexes, individuals or body sites, instead we found their level of uniqueness to vary by body site and age for both males and females. In combination with existing histological, chemical and behavioural analyses, our results indicate the presence of encoded chemical signals of age-sex and individual identity in cutaneous glandular secretions of brown bears. This study addresses an ecological knowledge gap for bears which can contribute towards further understanding of mating systems and social behaviour.

## Supplementary Information

Supplementary Information.

## Data Availability

Data and code is available at https://github.com/clw224/Clapham_etal_2023_BrownBear_VOC.
