# Supplementary material for: Brown bear skin-borne secretions display evidence of individuality and age-sex variation
Source: Sci Rep. 2023 Feb 23;13:3163. doi: 10.1038/s41598-023-29479-y (PMC9950453; doi:10.1038/s41598-023-29479-y)
Supplement: Supplementary file 1 — Supplementary Information. [file 41598_2023_29479_MOESM1_ESM.docx]

**Supplementary Information**

**Brown bear skin-borne secretions display evidence of individuality and age-sex variation**

Melanie Clapham^*^, Abbey E. Wilson^*^, Candace L. Williams & Agnieszka Sergiel

^*^ Co-first authors

File includes:

Supplementary Table S1-S8

Supplementary Figure S1 and S2

Table S1. The variance across the different groups of interest (body site, individual, sex, age class, and age-sex class) for compounds identified in samples collected from brown bears (unbalanced dataset) was significantly different.

|  | Body site (ID(Sex)) | ID(Sex) | Sex | Age class | Age*Sex |
| --- | --- | --- | --- | --- | --- |
| Bray-Curtis | < 0.0001* | < 0.0001* | 0.039* | 0.0014* | <0.0001* |
| Jaccard | < 0.0001* | < 0.0001* | 0.060 | 0.0017* | <0.0001* |

*Significant, P < 0.05, Betadisper with Bonferroni correction (R, *vegan* package).

Table S2. PERMANOVA output for Bray-Curtis and Jaccard distances, using full (unbalanced) and subsampled (balanced) datasets.

|  | Bray-Curtis (P-value) | | Jaccard (P-value) | |
| --- | --- | --- | --- | --- |
|  | Unbalanced | Balanced | Unbalanced | Balanced |
| Age | 0.000667 | 0.017250 | 0.001667 | 0.020375 |
| Sex | 0.051000 | 0.017250 | 0.054000 | 0.016167 |
| Age*Sex | 0.000250 | 0.000500 | 0.000250 | 0.000250 |
| ID(Sex) | 0.000250 | 0.000500 | 0.000250 | 0.000250 |
| Body site(ID(Sex)) | 0.487900 | 0.546000 | 0.537600 | 0.602700 |

P-values were adjusted by FDR.

Table S3. Compounds (mass@retention time) that contributed to the variation (≥2%) in the volatile profile within individuals nested within sex.

| 73.0@21.80 | 88.0@23.07 | 88.0@26.50 |
| --- | --- | --- |
| 88.0@26.37 | 72.0@26.39 | 2,5-Dichlorobenzyl alcohol |
| 74.0@24.95 | 88.0@24.87 | 62.0@25.27 |
| 72.0@1.97 | 74.0@24.96 | 84.0@21.14 |
| 88.0@26.36 | 60.0@14.51 | 86.0@24.84 |
| Boric acid, trimethyl ester | 88.0@23.48 | Benzaldehyde |
| 73.0@20.56 | 88.0@24.85 | 74.0@25.31 |
| 88.0@24.79 | 60.0@14.96 |  |

Table S4. P-values from both incidence and abundance generated by PERMANOVA for age-sex classes.

|  | Mature Male | | Mature Female | | Young Male | | Young Female | |
| --- | --- | --- | --- | --- | --- | --- | --- | --- |
|  | ID | Body site(ID) | ID | Body site(ID) | ID | Body site(ID) | ID | Body site(ID) |
| Bray- Curtis | >0.05 | >0.05 | 0.0004 | >0.05 | 0.0002 | >0.05 | 0.0196 | >0.05 |
| Jaccard | >0.05 | >0.05 | 0.0004 | >0.05 | 0.0002 | >0.05 | 0.0218 | >0.05 |
| *Significant, P < 0.05. | | | | | | | | |

Table S5. Compounds (mass@retention time) that contributed to the variation (≥2%) in the volatile profile within age classes separated by individuals nested within sex.

|  | Young: ID(Sex) | | Mature: ID(Sex) | |
| --- | --- | --- | --- | --- |
| Compound | p-value | SIMPER% | p-value | SIMPER% |
| 2,5-Dichlorobenzyl alcohol | > 0.05 | 2.1 | -- | -- |
| 72.0@26.39 | > 0.05 | 2.5 ± 0.08 | -- | -- |
| Boric acid, trimethyl ester | > 0.05 | 2.7 ±0.22 | -- | -- |
| 73.0@20.56 | 0.018* | 2.6 ± 0.25 | -- | -- |
| 73.0@21.80 | < 0.001* | 4.7 ± 0.34 | -- | -- |
| Benzaldehyde | -- | -- | > 0.05 | 2.0 |
| 62.0@25.27 | > 0.05 | 2.2 | > 0.05 | 2.1 |
| 88.0@26.50 | 0.014* | 2.2 | -- | -- |
| 60.0@14.51 | -- | n/a | > 0.05 | 2.2 |
| 88.0@26.36 | > 0.05 | 2.5 ± 0.14 | > 0.05 | 2.4 |
| 74.0@24.96 | -- | -- | > 0.05 | 2.5 |
| 74.0@24.95 | > 0.05 | 2.9 ± 0.16 | > 0.05 | 2.5 |
| 88.0@26.37 | -- | -- | > 0.05 | 3.2 |
| 84.0@21.14 | -- | -- | > 0.05 | 2.2 |
| 72.0@1.97 | > 0.05 | 2.6 ± 0.16 | > 0.05 | 3.2 ± 0.44 |
| 60.0@14.96 | > 0.05 | 2.2 ± 0.05 | -- | -- |
| 88.0@23.07 | > 0.05 | 2.1 | > 0.05 | 2.7 ± 0.26 |
| 88.0@23.48 | > 0.05 | 2.2 ± 0.05 | -- | -- |
| 88.0@24.79 | > 0.05 | 2.2 ± 0.06 | > 0.05 | 2.9 ± 0.14 |
| 88.0@24.87 | > 0.05 | 2.13 | > 0.05 | 2.4±0.30 |

*Significant, P < 0.05.

Table S6. Compounds (mass@retention time) present in number of samples with respect to age and body site within females. One unique (bolded), 21 signature dominant, and 5 dominant compounds were detected in samples collected from body sites within mature and young female bears.

|  | Mature | | | | Young | | | |
| --- | --- | --- | --- | --- | --- | --- | --- | --- |
| Compound | Cheek (n=4) | Flank (n=4) | Hump (n=4) | Pedes (n=4) | Cheek (n=3) | Flank (n=3) | Hump (n=3) | Pedes (n=3) |
| 88.0@23.74 | 4 | 3 | 3 | 1 | 1 | 1 | 1 | 1 |
| 88.0@21.94 | 3 | 3 | 2 | 3 | 3 | 2 | 0 | 1 |
| 72.0@26.59 | 1 | 4 | 2 | 0 | 0 | 0 | 0 | 0 |
| Benzaldehyde, 2,6-dichloro- | 1 | 0 | 0 | 2 | 2 | 3 | 1 | 0 |
| 104.0@1.68 | 0 | 1 | 1 | 0 | 0 | 3 | 2 | 0 |
| 62.0@24.95 | 2 | 2 | 3 | 0 | 2 | 3 | 2 | 2 |
| 59.0@24.98 | 0 | 2 | 3 | 1 | 2 | 3 | 1 | 0 |
| 59.0@1.55 | 1 | 0 | 0 | 1 | 0 | 3 | 2 | 2 |
| 62.0@1.96 | 1 | 0 | 2 | 2 | 1 | 3 | 2 | 0 |
| 60.0@25.34 | 1 | 0 | 2 | 0 | 1 | 3 | 0 | 2 |
| 93.0@1.49 | 0 | 1 | 1 | 2 | 1 | 3 | 1 | 1 |
| 57.0@25.59 | 0 | 0 | 0 | 1 | 1 | 3 | 1 | 2 |
| **62.0@2.63** | 0 | 0 | 0 | 0 | 0 | 3 | 0 | 0 |
| 92.0@4.53 | 0 | 0 | 0 | 0 | 0 | 3 | 1 | 0 |
| 62.0@2.30 | 0 | 1 | 0 | 0 | 0 | 0 | 3 | 0 |
| 92.0@3.33 | 1 | 0 | 0 | 0 | 0 | 0 | 3 | 0 |
| 88.0@22.60 | 3 | 2 | 2 | 4 | 2 | 2 | 0 | 2 |
| 72.0@1.97 | 1 | 2 | 1 | 4 | 2 | 2 | 2 | 1 |
| 59.0@1.79 | 2 | 2 | 1 | 3 | 1 | 2 | 0 | 3 |
| 88.0@25.02 | 2 | 2 | 1 | 0 | 1 | 2 | 0 | 3 |
| 104.0@2.66 | 0 | 0 | 0 | 2 | 1 | 0 | 0 | 3 |
| 88.0@23.30 | 3 | 4 | 2 | 3 | 3 | 2 | 2 | 2 |
| 88.0@29.18 | 0 | 2 | 4 | 2 | 3 | 1 | 1 | 2 |
| 88.0@23.15 | 1 | 4 | 3 | 4 | 3 | 2 | 2 | 1 |
| 88.0@23.59 | 4 | 4 | 3 | 3 | 2 | 3 | 3 | 2 |
| 88.0@22.72 | 1 | 4 | 2 | 4 | 3 | 2 | 1 | 3 |

Table S7. Compounds (mass@retention time) present in number of samples with respect to age and body site within males. Zero unique, 17 signature dominant, and 4 dominant compounds were found in samples collected from body sites within mature and young male bears.

|  | Mature | | | | Young | | | |
| --- | --- | --- | --- | --- | --- | --- | --- | --- |
| Compound | Cheek (n=3) | Flank (n=3) | Hump (n=3) | Pedes (n=3) | Cheek (n=10) | Flank (n=10) | Hump (n=9) | Pedes (n=8) |
| 88.0@21.49 | 3 | 0 | 1 | 0 | 1 | 0 | 0 | 3 |
| 86.0@25.11 | 3 | 1 | 0 | 1 | 1 | 1 | 2 | 0 |
| 88.0@24.88 | 1 | 3 | 1 | 1 | 5 | 5 | 5 | 4 |
| 88.0@26.42 | 0 | 3 | 2 | 1 | 3 | 3 | 7 | 4 |
| 62.0@25.32 | 1 | 3 | 2 | 2 | 2 | 2 | 1 | 0 |
| 62.0@25.51 | 0 | 3 | 1 | 0 | 1 | 2 | 3 | 1 |
| Benzaldehyde | 1 | 1 | 3 | 1 | 5 | 2 | 1 | 0 |
| 88.0@22.59 | 1 | 0 | 3 | 1 | 7 | 7 | 5 | 6 |
| 74.0@25.76 | 1 | 0 | 3 | 1 | 2 | 0 | 4 | 0 |
| 58.0@25.56 | 1 | 1 | 3 | 2 | 2 | 0 | 3 | 0 |
| 59.0@25.77 | 2 | 0 | 3 | 1 | 1 | 3 | 1 | 0 |
| 56.0@2.76 | 0 | 0 | 3 | 0 | 1 | 1 | 0 | 0 |
| 72.0@25.00 | 0 | 0 | 3 | 1 | 2 | 2 | 0 | 0 |
| 59.0@25.33 | 0 | 0 | 3 | 0 | 0 | 2 | 1 | 0 |
| 74.0@25.37 | 1 | 2 | 3 | 2 | 1 | 1 | 0 | 0 |
| 72.0@25.07 | 1 | 2 | 1 | 3 | 3 | 4 | 4 | 5 |
| 56.0@2.20 | 0 | 0 | 0 | 3 | 1 | 0 | 0 | 0 |
| 72.0@1.96 | 3 | 3 | 2 | 2 | 3 | 5 | 2 | 3 |
| 62.0@1.94 | 1 | 1 | 3 | 3 | 3 | 3 | 4 | 2 |
| 74.0@24.94 | 3 | 3 | 3 | 2 | 1 | 2 | 0 | 0 |
| 59.0@1.79 | 3 | 2 | 3 | 3 | 4 | 3 | 0 | 4 |

Table S8. Compounds (mass@retention time) present in number of samples with respect to individual. Zero unique, ten signature dominant, and one dominant compound were found across individuals.

| Compound | Y742 (n=8) | Y743 (n=4) | Y860 (n=8) | Y861 (n=8) | Y862 (n=7) | Y863 (n=8) | Y864 (n=8) | Y865 (n=4) | Y866 (n=8) | Y867 (n=8) | Y868 (n=4) |
| --- | --- | --- | --- | --- | --- | --- | --- | --- | --- | --- | --- |
| 74.0@24.94 | 8 | 1 | 0 | 0 | 0 | 0 | 1 | 0 | 2 | 4 | 3 |
| 59.0@24.99 | 2 | 4 | 2 | 4 | 4 | 3 | 6 | 2 | 1 | 5 | 2 |
| 60.0@25.24 | 1 | 4 | 0 | 0 | 0 | 0 | 0 | 0 | 1 | 1 | 2 |
| 88.0@23.29 | 2 | 1 | 8 | 7 | 6 | 6 | 7 | 3 | 4 | 6 | 1 |
| 88.0@23.57 | 2 | 0 | 8 | 7 | 3 | 4 | 6 | 3 | 4 | 6 | 0 |
| 88.0@24.81 | 3 | 1 | 8 | 6 | 2 | 7 | 3 | 3 | 3 | 1 | 0 |
| 86.0@25.01 | 6 | 0 | 3 | 2 | 3 | 1 | 3 | 1 | 3 | 6 | 4 |
| 59.0@1.79 | 6 | 0 | 3 | 1 | 0 | 6 | 2 | 1 | 5 | 5 | 4 |
| 74.0@25.24 | 4 | 1 | 0 | 0 | 0 | 0 | 0 | 0 | 2 | 7 | 4 |
| 74.0@25.37 | 4 | 3 | 0 | 0 | 0 | 0 | 0 | 0 | 1 | 2 | 4 |
| 72.0@1.96 | 6 | 1 | 5 | 3 | 0 | 3 | 3 | 4 | 5 | 5 | 4 |


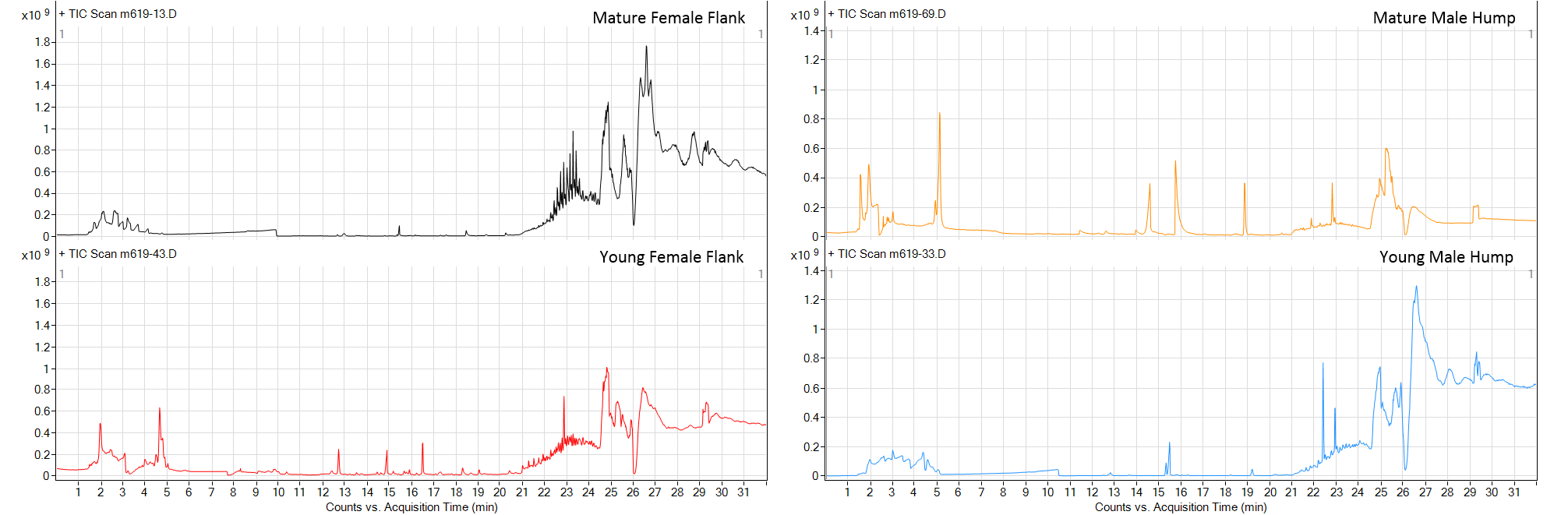


Figure S1. Representative total ion chromatograms of brown bear samples. The flank region in young females had the greatest number of signature compounds compared to all body sites and age classes within females. The hump region in mature males had the greatest number of signature compounds compared to all body sites and age classes within males.


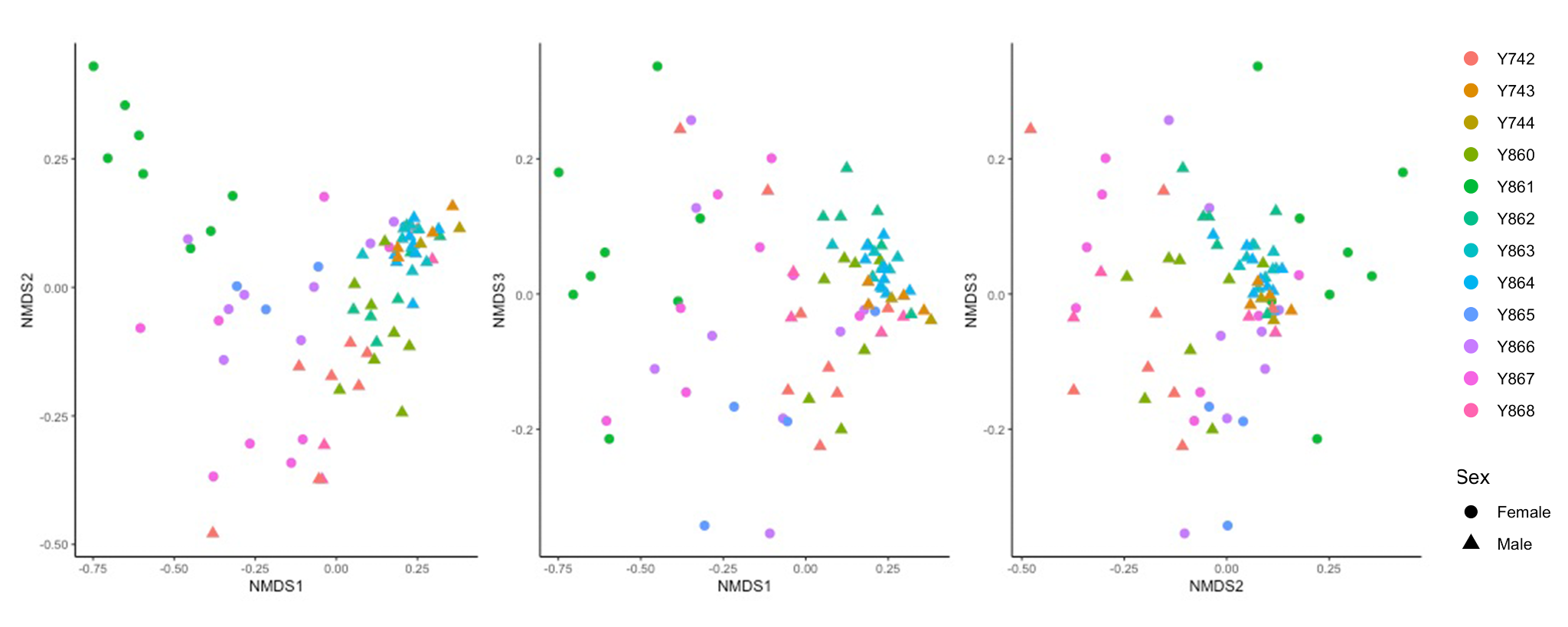


Figure S2. Nonmetric multidimensional scaling (nMDS) analysis displaying overall differences in volatile organic compounds as observed by mass spectrometry based on Bray-Curtis distances (stress: 0.13, Goodness of fit R^2^: 0.987).
